# Experiences and challenges in the health protection of medical teams in the Chinese Ebola treatment center, Liberia: a qualitative study

**DOI:** 10.1186/s40249-018-0468-6

**Published:** 2018-08-16

**Authors:** Ying Li, Huan Wang, Xu-Rui Jin, Xiang Li, Michelle Pender, Cai-Ping Song, Sheng-Lan Tang, Jia Cao, Hao Wu, Yun-Gui Wang

**Affiliations:** 1 Department of Social Medicine and Health Service Management, Army Medical University (Third Military Medical University), Chongqing, China; 2 Bureau of Medical Affairs Administration, First affiliated Hospital, Army Medical University (Third Military Medical University), Chongqing, China; 3 The Cadet Brigade of Clinic Medicine, Army Medical University (Third Military Medical University), Chongqing, 400038 China; 4 Duke Global Health Institute, Duke University, Durham, NC USA; 5 Bureau of nurse Administration, Second affiliated hospital, Army Medical University (Third Military Medical University), Chongqing, China; 6 Department of Hygienic Toxicology, Army Medical University (Third Military Medical University), Chongqing, China; 7 Army Medical University (Third Military Medical University), No. 30 Gaotanyan Road, Shapingba district, Chongqing, 400038 China

**Keywords:** Infectious diseases, Ebola, China, Ebola treatment center, Medical team, Liberia

## Abstract

**Background:**

Health care workers are at the frontline in the fight against infectious disease, and as a result are at a high risk of infection. During the 2014–2015 Ebola outbreak in West Africa, many health care workers contracted Ebola, some fatally. However, no members of the Chinese Anti-Ebola medical team, deployed to provide vital medical care in Liberia were infected. This study aims to understand how this zero infection rate was achieved.

**Methods:**

Data was collected through 15 in-depth interviews with participants from the People’s Liberation Army of China medical team which operated the Chinese Ebola Treatment Center from October 2014 to January 2015 in Liberia. Data were analysed using systematic framework analysis.

**Results:**

This study found numerous bio-psycho-socio-behavioural risk factors that directly or indirectly threatened the health of the medical team working in the Chinese Ebola Treatment Center. These factors included social and emotional stress caused by: (1) the disruption of family and social networks; (2) adapting to a different culture; (3) and anxiety over social and political unrest in Liberia. Exposure to Ebola from patients and local co-workers, and the incorrect use of personal protective equipment due to fatigue was another major risk factor. Other risk factors identified were: (1) shortage of supplies; (2) lack of trained health personnel; (3) exposure to contaminated food and water; (4) and long working hours. Comprehensive efforts were taken throughout the mission to mitigate these factors. Every measure was taken to prevent the medical team’s exposure to the Ebola virus, and to provide the medical team with safe, comfortable working and living environments. There were many challenges in maintaining the health safety of the team, such as the limited capability of the emergency command system (the standardized approach to the command, control, and coordination of an emergency response), and the lack of comprehensive international protocols for dealing with emerging infectious disease pandemics.

**Conclusions:**

The comprehensive and multidisciplinary measures employed to protect the health of the medical team proved successful even in Liberia’s resource-limited setting. The global health community can learn valuable lessons from this experience which could improve the safety of health care workers in future emergencies. These lessons include: establishing capable command systems; implementing effective coordination mechanisms; providing adequate equipment; providing training for medical teams; investing in the development of global health professionals; and improving research on ways to protect health care workers.

**Electronic supplementary material:**

The online version of this article (10.1186/s40249-018-0468-6) contains supplementary material, which is available to authorized users.

## Multilingual abstracts

Please see Additional file 1 for translations of the abstract into the five official working languages of the United Nations.

## Background

Health care workers (HCWs) are at the forefront of the battle against infectious diseases, placing them at a greater risk of infection [1, 2]. For example, a reported 419 HCWs have contracted Viral hemorrhagic fevers (VHFs) within health care facilities, of whom 97 (23.15%) have died [3]. The first reported nosocomial Crimeane Congo hemorrhagic fever (CCHF) outbreak in Pakistan in 1976 resulted in 10 infected HCWs [4]. During the outbreak of Ebola Hemorrhagic Fever in Uganda between August 2000 and January 2001, 14 of the 22 (64%) HCWs in Gulu were infected [5]. Three HCWs were infected during the outbreak of Ebola hemorrhagic fever in the Republic of the Congo in 2003 [6]. In another outbreak in Uganda during 2007–2008, 14 of the 192 infected people were HCWs [7]. These high rates of infection suggest that HCWs lack sufficient knowledge about clinical presentation and infection control measures of VHFs [8], and also lack appropriate protective equipment.

Ebola virus disease (EVD) threatened global health security when an outbreak occurred in West Africa in 2014 [9]. By June 2016, a total of 28 616 confirmed, probable and suspected EVD cases had been reported in Guinea, Liberia and Sierra Leone, resulting in 11 310 deaths by 10 June 2016 [10]. EVD spreads through direct contact with bodily fluids [11]. This transmission mode, and HCWs’ frequent contact with infected patients place HCWs at a high risk of infection. Incidence of EVD among HCWs was 103-times higher than that of the general population between May 23 and Oct 31, 2014 [12]. By May 20, 2015, 869 HCWs were infected, and 507 HCWs had died in West Africa [13]. HCWs have the highest levels of crude mortality rates, and the mortality rate of HCWs in Sierra Leone was as high as 80% (as of 26 October 2014) [14]. HCWs EVD infection was not limited to West Africa, but spread with the disease to the United States and Spain [15, 16]. High rates of HCWs’ EVD infection were reported in Ebola treatment centers (ETCs) in West Africa. By August 14, 2014, approximately 36% of the infected HCWs worked in ETCs in Liberia [9]. Preventing EVD and other infections among HCWs is crucial to improving future public health responses around the world [17, 18].

At the beginning of September 2014, the People’s Liberation Army (PLA) sent three Chinese military medical teams (CMMTs) totalling more than 500 persons to Sierra Leone and Liberia to help with the EVD pandemic. The CMMTs in these two countries treated a total of 894 patients, including 297 confirmed cases of EVD [19]. The Chinese ETC carried out the following activities in Liberia within a sixth-month mission: (1) patient observation; (2) diagnosis and treatment (177 suspected, probable, or confirmed EVD cases, and 60% of confirmed Ebola patients were cured); (3) trained 1520 local HCWs, peace-keeping police and community members; (4) and provided guidance on infectious disease control for staff in the Chinese Embassy and other Chinese enterprises.

Despite, the high rates of EVD in Sierra Leone and Liberia, HCWs working in the Chinese ETCs located in these countries had a “zero infection” rate [20, 21]. Examining how this remarkable achievement was accomplished is important to establishing successful policies and procedures for other diseases and other locations.

Although studies have previously reported on various mental health [22] and physical measures taken by Chinese ETCs to protect HCWs from EVD infection [23, 24], these studies were all conducted in China, and only reported on measures taken during the mission, and did not report on measures taken before and after the mission. No study has yet analysed the full spectrum of bio-psycho-social risk factors to which HCWs are exposed throughout their mission. Neither has the existing literature comprehensively examined the policies and procedures used to protect the overall health of medical teams. This study aims to understand how China successfully managed to protect their medical team, and use this experience to identify recommendations to improve the global response to emerging and re-emerging infectious diseases.

## Methods

### Study design

This was a qualitative study, involving semi-structured, in-depth interviews with the first elite military PLA medical team deployed to Liberia. We examined the factors that placed the medical team’s health at risk, and the measures taken to protect the medical team during all three phases of their mission: before-deployment; during-deployment; and post-deployment. We used the bio-psycho-social health model to examine the risk factors and protection policies/procedures put in place by the medical team in each of these phases. The bio-psycho-social health model was first developed by G.L. Engel in 1977 [25]. This model proposes that health is the interaction of biological factors (genetic, biochemical, etc), psychological factors (mood, personality, behaviour, etc.), and social factors (cultural, familial, socioeconomic, medical, etc.). These factors interact with one another and function as both protective and risk factors for health and disease. The World Health Organization (WHO) defines health as a “state of complete physical, mental, and social well-being, and not merely the absence of infirmity” [26]. As such, a comprehensive approach to achieving health should be multidisciplinary, and should include physical, mental and social aspects of health. This study explores the factors associated with the medical team’s health from biological, psychological, and social perspectives.

### Study area and context

In Liberia, EVD was first reported in Lofa County on March 30, 2014 [27]. More than 10 500 cases of Ebola were reported in Liberia, causing nearly 5000 deaths [28]. WHO declared Liberia Ebola free in September of 2015 [29]. By August 14, 2014, approximately 36% of the infected HCWs worked in ETCs in Liberia [9]. The CMMT arrived in Liberia in September 2014 for a six-month mission. The CMMT composed of 163 multidisciplinary military experts including managers, epidemiologists, hygienists, medical technicians, psychologists, medical doctors, nurses, engineers, logistics support staff, and foreign affairs officials. Most of the military medical staff came from the Third Military Medical University in Chongqing, with a few dozen from other military hospitals in Shenyang. The team was based in the Chinese ETC located in Monrovia at the Samuel Kanyon Doe Sports stadium. The Chinese ETC was independently run by the Chinese Government, and was then formally transferred to the government of Liberia on May 13, 2015.

### Sample size and method

A purposive sampling method was used to select interview participants from diverse positions within the Liberian Chinese ETC. Selected participants included the Deputy Director of the Chinese ETC, the Director of the Bureau of Nursing in the Chinese ETC, clinical personnel, public health personnel, logistics support staff, psychologists, and foreign affairs officials. Many of these members had participated in the severe acute respiratory syndrome (SARS) pandemic in 2003, and had valuable experience and knowledge about pandemics that proved useful in the Ebola response.

### Data collection

Topic guides for the interviews were utilized to elicit information on risk factors during all three phases of the CMMT deployment. Topic guides had 4 sections. Section “Multilingual abstracts” collected the socio-demographic characteristics of the respondents (gender, age, role in the medical team, professional title, and previous experience working in infectious disease or providing medical services during peacekeeping missions). Section “Background” composed of questions on perceived biological, psychological, and social risk factors that threatened the medical team’s health before-deployment, during-deployment, and post-deployment. Section “Methods” covered health protection policies and procedures for the medical team (health, social or diplomatic measures taken to protect the health of medical team before-deployment, during-deployment, and post-deployment). Section “Results” included challenges (any factor directly or indirectly impacting the health of the medical team, as well as challenges for the overall Ebola control efforts). All interviews were audio recorded with participant consent and lasted around 60 min. All interviews took place between October 4, 2015 and April 22, 2016, and were conducted in Chinese. A teacher (YL), graduate student (HW) and two undergraduate students (XJ and XL) collected data after they received data collection training.

### Data analysis

All interviews were transcribed and carefully reviewed for accuracy. We analysed all interviews using the thematic framework approach [30, 31]. The thematic framework approach to qualitative data analysis is a five-step process that involves: (1) *familiarization* with transcripts, (2) *developing a theoretical framework* based on interview guidelines, and recurrent and important themes in individual transcripts, (3) *coding* the transcripts using the framework, (4) *summarizing data in an analytical framework* to create brief summaries of participant responses, and (5) *data synthesis, and interpretation* to compare themes and sub-themes across interviews [30, 32]. Analyses of the interviews yielded three themes and seven sub-themes for each phase of the mission. These were divided into the following categories: risk factors (socio-psychological factors, behavioural factors and biological factors); protection measures (health measures, social measures and global health diplomacy actions); and challenges. We present the results according to the three phases of the mission.

## Results

### Socio-demographic characteristics of respondents

Fifteen medical team members were selected, ten men and five women (Table 1). The majority of participants (9/15) were aged 40–49 years, five members were aged 30–39, and one was aged 50 and over. More than half of the participants had clinical and public health credentials, two were senior medical technicians, and two were teachers from the English department at the Third Military Medical University (the teachers role was to assist with translation and communication). More than two thirds of the participants were senior health professionals. Notably, close to half of the participants had at least one previous assignment working in infectious disease, or providing medical services during a United Nations peacekeeping mission in Africa.

**Table 1:** The demographic characteristics of participants

Characteristics	Number
Gender	
Male	10
Female	5
Age	
30–39	5
40–49	9
≥ 50	1
Roles	
Hospital leaders	2
Clinical health worker	5
Public health workers	3
Logistics staffs (technicians, laboratory technicians)	2
Psychologist	1
Foreign officials	2
Professional title	
Middle	4
Associate senior	5
Senior	6
Times of previous experience	
None	8
1–2	5
≥ 3	2

### Health risk factors of the medical team

We divided the various health risk factors identified by interview participants by each phase of the mission (Table 2). All factors were classified as socio-psychological, behavioural or biological. These factors directly or indirectly impacted the health of the CMMT members to varying degrees.

**Table 2:** Risk factors faced by medical team

Phrases	Risk factors	Example citation
Phase one (before-deployment)	Socio- psychological factors 1. Disturbed social network due to closed training 2. Anxiety and fear about infection of diseases (EVD, malaria, HIV and TB) 3. Worry about heavy workload, the underdeveloped public health system, hard living environment, social chaos and unsafe social environment, and different cultural in Liberia	There are other epidemics such as cholera, yellow fever and malaria…. All felt anxiety because EVD is deadly disease and the mortality of patients is high *(General head nurse in ETC)*
Phase two (during-deployment)	Socio- psychological factors 1. Disturbed social network as separating from their families and friends 2. Cultural differences 3. Unsafe living environment like anopheles, unsafe food and water and social chaos 4. Shortage of food and health resources (medical supplies and professionals to run ETC) 5. Anxiety and fear about infection of diseases (EVD, malaria, TB and HIV) 6. Worry about complex PPE dressing 7. Worry about families where they were unable to look after them if needed	Social and political unrest in local place make we feel unsafe. *(Foreign affairs official in ETC)* The local fruits and water are possibly contaminated with Ebola virus. The fruit like banana is very good. But we feared to eat local fruits or drink local water. It is difficult to know whether the banana is contacted by money with Ebola virus. *(Leader of logistics support group in ETC)* HCWs met patients every day to provide health care such as transfusion. So they felt pressure. They worry about separating from families whom they could not look after when needed. They need to get used to local living environment. *(Psychological experts)* HCWs who work for long time with PPE would sweat a lot and felt uncomfortable. *(General head nurse in ETC)*
	Behavioural factors 1. Contacting with local patients as providing care for them 2. Recruiting and training local people to work in ETC 3. Incorrect operation with PPE due to fatigue	During providing healthcare for patients, HCWs easily forget self-health protection. We recruited local people to work in ETC due to shortage of personnel. Those local people lived in different communities in different area. We cannot know whether their families and friends have risk of infection. Local people were paired with PLA HCWs which would increase risk of infection *(Deputy director of ETC)*
	Biological factors 1. Susceptible to infectious disease in Liberia due to living environment which included mosquitoes, possible exposure to contaminated food and water	Local epidemics, such as cholera, yellow fever, malaria, tuberculosis. We all are susceptible to those epidemics in Liberia. *(General head nurse in ETC)*
Phase three (Post-deployment)	Socio- psychological factors 1. Isolated living environment to prevent them from infection or transmission 2. Inconvenient communicate with colleagues and families 3. Worry about infection of diseases	We could not meet with families and friends during 21-day’s medical isolation. *(Clinical doctor in ETC)* Nobody knows whether we are infected with any infectious diseases. We still worry about the risk of infection. Our social network is still disturbed. It is possible to have psychological influence. *(Psychological experts in ETC)*
	Biological factors Physical fatigue after long time hard work	We felt very fatigue after long time hard work in Liberia and long travel from Liberia back to China. *(General head nurse in ETC)*

Notes: *EVD* refers to Ebola virus disease, *HIV* refers to Human Immunodeficiency Virus, *TB* refers to tuberculosis, *ETC* refers to Ebola treatment centre, *HCWs* refers to healthcare workers, *PPE* refers to personal protection equipment, *PLA* refers to People’s Liberation Army

#### Phase one (before-deployment)

Participants identified several socio-psychological and behavioural risk factors during this phase. Many of the participants reported experiencing disruption to their social and family networks as a result of the required 6 weeks training camp in preparation for deployment. This led to feelings of isolation and caused separation anxiety, negatively impacting the CMMT’s mental health. Apprehension about moving to a new country, and living in a different culture also caused anxiety. All participants reported feeling fear and anxiety about their exposure to Ebola or other infectious diseases (HIV [human immunodeficiency virus], malaria and tuberculosis), as well as the arduous mission they would have to undertake in Liberia. All participants worried about the difficulties they would face working in the underdeveloped local public health system in Liberia, as well as enduring difficulties associated with living in a different environment. Approximately half of the participants (7/15) felt nervous about living and working in Africa based on their perceptions about armed conflict, and social and political unrest in the region. Participants with previous experience working in Africa were apprehensive about returning to what they knew would be a difficult living and working environment. One individual described feeling pressure and anxiety due to the fact that he had no previous experience with such a complex international mission. All participants reported that they found the training intensive.

#### Phase two (during-deployment)

More complicated factors threatened the health of medical team during this phase.

##### Socio-psychological factors

Most of the participants identified the following risk factors to their mental health as a result of their new social environment: the disruption caused by leaving behind their family and friends; being immersed in a different culture; witnessing social and political unrest; dealing with food shortages, and lack of medical supplies; and dealing with a shortage of trained health personnel to work in the ETC. Most of the participants experienced anxiety and fear about contracting infectious diseases (EVD, malaria and HIV), and about correctly using personal protective equipment (PPE). Concern for their own welfare was exacerbated by anxiety about their families’ welfare, and being too far away to help them if any emergencies arose.

##### Behavioral risk factors

Notably, key informant interviews with health leaders emphasized the shortage of medical supplies, especially the shortage of PPE, which resulted in the re-use of PPE, a practice that increased the risk of Ebola infection among HCWs even if the PPE was sterilized. The majority of participants believed that providing care for local patients, and training local HCWs substantially increased their risk of infection. Participants stated that there was incorrect use of PPE due to fatigue, further increasing the risk of infection. The medical team hired local HCWs to help in the ETC. Six participants thought that working alongside local HCWs would increase their risk of infection, since local HCWs might carry infectious diseases into the ETC after contact with infected families or community members. However, one participant argued that local HCWs might reduce the risk of infection because they could communicate with patients easily, help the CMMT to communicate with patients, and ensure adherence to medical protocols. (iii) ***Biological factors:*** all participants recognized that their new living environment, exposure to mosquitoes, and possible exposure to contaminated food and water placed them at an increased risk of infection from local infectious diseases.

#### Phase three (post-deployment)

##### Socio-psychological risk factors

After their mission was completed, all CMMT members were required to be quarantined for 21 days for medical observation, as Ebola has a 21 day incubation period. During this time they were not permitted contact with each other in order to avoid cross infection. Most of the members found it difficult to communicate with colleagues and families during this period. Furthermore, they were not sure if they were infected with EVD or not, and this uncertainty caused anxiety.

##### Biological factors

Team members suffered from fatigue after their long, physically, emotionally and mentally draining mission in Liberia. This fatigue lowered their resistance to disease.

### Health protection policies and procedures for the medical team

Prior to deployment (Table 3), almost all of the participants mentioned that the CMMT had modified the existing PPE, and strengthened the PPE protocols and procedures based on suggestions and guidelines from WHO and Chinese Center of Disease Control and Prevention (China CDC). For example, additional protective equipment such as disposable shoulder caps, boots, and waterproof clothes were added to the standard PPE. Three-layers of PPE were used (two more layers than the standard WHO recommendation, and one more layer than the standard China CDC recommendation). This third layer of PPE added one more protective barrier during interaction with Ebola patients, but did not pose any additional risk when correctly removed according to standard protocols. All personnel in the Chinese ETC were required to attend a 6 week rigorous training course on Ebola, and the correct PPE protocol. Participants reported that members of the CMMT had been carefully selected for their expertise and experience, and over two-thirds of them had previous experience working with infectious diseases in Africa. For example working during the SARS pandemic, and providing medical care during international peacekeeping missions or joint military exercises. These experiences better enabled members of the team to cope with stress, unfamiliar environments, and basic living conditions in Liberia. Mental health services, vaccines, and medications were available to CMMT members throughout their mission, a factor considered important by all participants. Key informants and participants in charge of foreign affairs reported that every effort was made to learn more about the local geographical and social environment, EVD epidemic, local health system, and culture in Liberia. Notably, some participants found that social measures such as the Third Military Medical University providing care for their families in China while they were on deployment helped to sooth their anxiety about leaving their families for such a long period.

**Table 3:** Health protection measures before-, during and post-deployment

Measures		Example citation
Before-deployment		
Health measures	1. To carefully select team members 2. To provide PPE of high quality and make standard protocols for PPE use 3. To organize 1.5 month intensive training on procedures of Ebola diagnosis and treatment, PPE use, prevention and surveillance as well as English 4. To provide mental health support to relieve anxiety 5. To provide vaccine, medicine to increase the immunity, health surveillance for all team members 6.To strengthen physical exercise	Firstly, the medical team had been carefully selected and over two-thirds had previous experience with infectious diseases and Africa. Secondly, we took active prevention such as taking vaccines, taking drugs to prevent cholera, yellow fever and malaria- and so on. Besides, physical exercise was strengthened for all medical teams. *(Deputy director of ETC)* We modified PPE and developed more stringent PPE protocols and procedures based on suggestions and guidelines from WHO and the CDC of China.(*Leader in public health group in ETC*)
Social measures	To provide help with family life events and care for team member’s children and the elderly if necessary	Leaders in our institute see our families to learn about any need of health which helped to sooth their concerns about their families *(Public health personnel)*
Health diplomacy	1. To visit the stadium in Monrovia and existing ETCs from other countries, and to consult with personnel in NECC and local Liberian who had study experience in China to learn more about the local physical and social environment, epidemic, health system and culture etc.in Liberia 2. To consult with Chinese peacekeepers back from or still in Liberia to learn local epidemic	A group with 5 experts from this PLA medical team visited the stadium in Monrovia, contacted with local government, the presidents and NECC in Liberia to learn the situation of Ebola control. *(Leader of foreign affairs office in ETC)* We also invited Chinese peacekeepers who worked in Liberia before and Chinese experts from CDC to give lectures about Liberia to all medical team *(Leader of logistics support group in ETC)*
During-deployment		
Health measures	1. To construct ETC with strict layout of three zones and two routes and to equip it with advanced a distinctive communication system(audio loud speakers, video monitoring system and ward calling system) 2. To establish and implement a three-level safety supervision program (the first level supervision---mutual supervision between paired members; the second-level supervision ---daily supervision by the medical personals on duty; the third-level supervision---hospital-level supervision by a 10-member expert team) for all staff 3. To implement strict environmental cleanness and disinfection 4. To set up expert team to provide health service for all team members 5. To recruit the experienced local people and local people who can speak Chinese to help with communication 6. To train the newly recruited local people and group them with PLA medical team 7. To provide lectures about doctor-patient communication before receiving patients in ETC 8. To continue training on procedures of diseasediagnosis and treatment, prevention and surveillance and PEE use 9. To wear PEE, diagnose and treatment patients following the strict protocol 10. To give professional psychological counselling and health care for team member if necessary	Red zone (contaminated zone) and green zone (clean zone) were required internationally for ETC construction. We constructed ETC with one more zone, the semi-contaminated zone marked with yellow. We equipped ETC with video system to monitor operation during care for patients which could prevent HCWs infection. We recruited and trained local skilled people work in China ETC. We also recruited one local people who can speak Chinese, had background of medical education and could help HCWs to communicate with patients. *(Foreign affairs official in ETC)* Unique health protection measures included strict protocol for PPE use, paired work group, daily supervision by on duty medical professionals, video and voice monitor system. The whole process of PPE dressing was watched and guided by professionals through video and voice system to minimize the opportunities of HCW exposure to Ebola. *(Deputy director of ETC)* Medical team need some time to adapt to local environment at the beginning. We provide psychological service to relax them. Lectures about doctor-patient communication before receiving patients were provided for medical team. We refined ETC ventilation and disinfection measures. *(Psychological experts in ETC)*
Social measures	1. To import food, equip ETC with air conditioners and mosquito killer, to purify drinking water by self 2. To have security team to ensure safety and reduce exposure to Ebola outside of workplace 3. To set up Weichat and telephone for team member communication with colleagues and families 4. To abide by the strict daily working and living schedule to ensure good rest 5. To provide care for team member’s children and the elderly if necessary 6. To launch paperless office to reduce contact with patients	We imported food for our medical team. We provided air conditioners and mosquito killer, drinking water through digging a well by ourselves and clean the water by machine we brought with. We controlled contact with local people in order to prevent from infection of infectious diseases. *(Leader of logistics support group in ETC)* We set 3 approaches for medical team including (such as Weichat, telephone) to help them with communication. *(Psychological experts in ETC)* We launched paperless office, for example, all patients’ diagnosis and treatment were recorded in the medical information system which prevented infection from patient’s contact. *( Senior medical technicians)*
Health diplomacy	1. To join the joint command system to get diseases surveillance information 2. To apply some essential supplies from the United Nations or local government 3. To coordinate with embassy, local Chinese enterprises to mitigate supplies shortage 4. To communicate with ETCs from other countries	We join the joint command system to get diseases surveillance information. This system would release updated information about local epidemics. *(Deputy director of ETC)* We applied essential supplies from UN or local Chinese enterprises through Chinese Embassy in Liberia. We also applied many essential supplies from local government. We also coordinate local Customs and Foreign affairs. *(Leader of logistics support group in ETC)* Managers actively participated in communication with ETC s from other countries. *(Leader of foreign affairs office in ETC)*
Post-deployment		
Health measures	1. To isolate all team members for 21 days 2. To organize experts to observe sign and symptoms 3. To provide nutritional diet 4. To provide psychological counsellors when necessary 5. To strengthen physical exercise	We could not contact with families, friends in the 21-day’s observation. We were provided with daily monitoring for signs and symptoms of Ebola or other infectious diseases. We have diet separately and the food is very good and nutritional. *(Clinic doctor in ETC)* During 21-day’s observation, we recover from fatigue due to hard work in Liberia. …There were nurses and doctors monitored our health every day. *(Nurse in ETC)* A lots of entertainments and psychological counsellors were provided for us. *(Leader of logistics support group in ETC)*.
Social measures	1. To provide comfortable rest and living conditions 2. To provide internet which would help with communication with friends and family	During observation, good living and rest conditions were provide for all medical team members. For this observation, we prepared a lot of things ahead of their come back from Liberia. *(General head nurse in ETC)* Internet was provided for everyone and they could access to internet anytime. Internet helped them to communicate with families and friends *(Public health personnel)*.

Notes: *HCWs* refers to health care workers, *PPE* refers to personal protection equipment, *WHO* refers to World Health organization, *CDC* refers to centre of disease control and prevention, *PLA* refers to People’s Liberation Army, *NECC* refers to National Ebola Command Centre, *EVD* refers to Ebola virus disease, *HIV* refers to Human Immunodeficiency Virus, *TB* refers to tuberculosis, *ETC* refers to Ebola treatment centre. UN refers to united nation

This study found that additional measures besides supplying high quality PPE were adopted to protect both the physical and mental health of all staff in the Chinese ETC (Table 3). The physical design of the Chinese ETC, and the strict protocols regarding movement around the building helped to prevent the spread of infection from patients. The layout included “three zones (the first “green” zone, was the non-contaminated zone, the second “yellow” zone was a semi-contaminated zone, and the third and final “red” zone was a contaminated zone).” Two routes were used to move between these zones (one “red” route used by inpatients and a separate “blue” route used by HCWs). Additionally, a distinctive communication system was used, HCWs were monitored by video surveillance, and if safety policies and procedures were not followed correctly, the individuals were alerted immediately to the problem over loud speakers. This Chinese ETC communication system worked effectively to supervise the operations of medical staff, and provide timely guidance to correct any safety breaches. The Chinese ETC also employed a three-level safety supervision system to ensure the correct and effective use of PPE, and diagnosis and treatment of EVD (the first-level supervision — mutual supervision between paired members; the second-level supervision — daily supervision by the medical personal on duty; the third-level supervision — hospital-level supervision by a ten-member expert team responsible for long-term inspection of ETC staff). These protocols together with strict disinfection measures were the corner stone of the protection strategy to prevent HCWs from EVD infection. In addition, an expert team was available to provide health services to all staff when necessary. The majority of participants reported that factors which might indirectly increase risk of infection with EVD were taken into consideration when developing the health protection policies and procedures. For example, local HCWs with experience working in ETCs run by other countries were recruited to alleviate the shortage of HCWs in the Chinese ETC. These HCWs were paired with the Chinese HCWs to ensure that they used their PPE correctly, and understood the zoning and routing systems.

Many social measures were also implemented to ensure a safe living and working environment. Lectures about doctor-patient communication were provided before receiving patients in ETC. The Chinese ETC engaged in certain health-related diplomatic activities which helped to provide a supportive environment for the medical team. For example, managers actively communicated with ETCs from other countries, and arranged to receive essential supplies from the United Nations or local Chinese enterprises established in Liberia.

During the 21 day quarantine after the mission, measures to ensure the physical and mental health of CMMT members included: providing active fever/symptom surveillance, ensuring members had a nutritional diet, making mental health counselors available to members when necessary, and ensuring members were given physical exercise. Additionally, to mitigate social risk factors, CMMT members were provided with comfortable living conditions and internet access to facilitate communication with friends and family outside (Table 3).

### Challenges

Interviews revealed a number of challenges that had a direct or indirect impact on the health of all the CMMT members, as well as the overall Ebola control efforts (Table 4). These challenges included:

**Table 4:** Challenges in health protection for medial team in China ETC in Liberia

Challenges	Example citation
1. The capability of emergency command system (CS) needs improvement	I think it is very important to establish a command system (CS) to command the battle to fight against EVD. This CS might promote effectiveness of all ETCs. For example, the CS could coordinate all ETCs to work effectively and consider interests of all ETCs. CS could coordinate all resources among ETCs in order to ensure all ETCs could run. CS could coordinate local transportation for materials for ETCs. For example, due to the different procedures required by customs in different countries, our materials were delayed to ETC when arrived in Liberia. For example, in order to treat that child who infected with EVD and was very severe, we need oxygen cylinder, our director of China ETC took a lot of time to get one for the child. Another example is the incinerator, we take a lot of time to coordinate it. *(Leader of foreign affairs office in ETC)* We got support from many Chinese enterprises in Liberia. We coordinated the materials through Embassy of the Chinese people’s Republic of China in Liberia. We also asked Ministry of Health in Liberia, UN. We also coordinated with custom and Foreign Office in Liberia for the transportation of materials for our ETC. *(Leader of logistics support group in ETC)*
2. International guidelines on how to respond to Ebola were not readily available	Up to deployment of our medical team, there is no standard or protocol for our medical team to refer to. In WHO website, there is only a special website to publish and update EVD epidemic, but no special website to publish any standard or protocol for international medical team to respond to EVD. We prepared our medical team with the experience from fighting SARS in China in 2003. But there are substantial differences between these two diseases, working and living environment in China and in Liberia. We found shortage of supplies and HCWs when we arrived in Liberia just because of no guidelines for preparations. *(Deputy director of ETC)*
3. No prepared medical teams for emergent infectious diseases and inadequate equipment deposit was another kind of challenge.	There need internationally prepared medical team for this emergent infectious diseases. The Global Foreign Medical Teams Registry has minimum standards for international health workers. Those standards also facilitate coordination between aid providers and recipients. The temporarily established medical teams are often unfamiliar with the international emergency response systems and standards and they might not know the usual coordination mechanisms. Besides, there lacked of registered factories to produce and deliver adequate PPE for HCWs. It is difficult to get PPE of high quality. *(Deputy director of ETC)* The prepared medical team should have a standard training camp. The training camp should simulate the living and work environment of the location they would be deployed to. If medical teams receive training in a similar environment they would have a better health when we arrive in field. *(Senior medical technicians)*
4. Participating in meeting for communication between ETCs increased risk to infection.	In affected countries, there should be a website for HCWs to communicate on experiences or lessons in responding to EVD in order to prevent medical team from infection with Ebola. But participating in meeting by NECC or visiting ETCs were the only approach for ETCs to communicate. In this case, this approach resulted in increasing contact with staffs from ETCs which might increase risk for contact and is not appropriate.*(Deputy director of ETC)*
5. Shortage of professionals who familiarize with health diplomacy challenged the medical team.	We lacked of diplomatic experience though we have experience to participating UN peacekeeping in Africa. Peacekeeping is different from anti-Ebola. We need more training for health diplomacy and we should cultivate more professionals *(Psychological experts)*.

*CS* refers to command system, *ETC* refers to Ebola treatment centres, *EVD* refers to Ebola virus disease, *HCWs* refers to healthcare workers, *UN* refers to United Nations, *SARS* efers to Severe Acute Respiratory Syndrome, *NECC* refers to National Ebola Command Centre

#### An inefficient emergency command system

The National Ebola Command Center (NECC) in Liberia was responsible for coordinating the Ebola response. Many international organizations and countries assisted Liberia in the global Ebola response. However, NECC’s lack of experience coordinating such a complex response caused complications which contributed to an increased risk of HCW infection. For example, a majority of participants reported that inefficient coordination with customs resulted in the delayed delivery of essential supplies for the ETC; inefficient coordination on the assignment of patients to ETCs meant that the Chinese ETC were not adequately informed about the number of patients expected to be admitted, or when they would arrive. Lack of logistical support on how to handle corpses, or waste products from ETCs also compromised infection prevention practices.

#### Comprehensive international guidelines on how to respond to Ebola were not readily available

Key informant interview participants disclosed that due to the lack of readily available comprehensive guidelines, the CMMT had difficulty estimating the supplies and personnel needed to run the 100-bed ETC in Liberia. This consequently resulted in a shortage of personnel and supplies. The CMMT relied on their previous experience with SARS in 2003, but this knowledge alone was not sufficient as the social and physical environments of China and Liberia are substantially different. Subsequently, 85 local people were recruited to work in the Chinese ETC to fill the human resource shortage, a practice that as mentioned earlier, many participants believed increased their risk of infection. Additionally, the CMMT was not provided with guidelines on how to prevent local diseases other than Ebola, or how to adjust to the local culture and environment, or how to contact local organizations when necessary. All of this made the CMMT less equipped for their mission, and might have directly or indirectly threatened their health.

#### Lack of readily prepared medical teams, and inadequate equipment were other major challenges

The Global Foreign Medical Teams Registry sets minimum standards for international health workers, and allows teams to clearly outline their services and skills in order to facilitate a more effective response and efficient coordination between aid providers and recipients. However, key informants reported that there is still an urgent need for a certain number of prepared international medical teams which can quickly respond to future global health crises like emergent infectious diseases. The medical teams organized are often unfamiliar with the international emergency response systems and standards, and may not be able to adapt smoothly to the standard coordination mechanisms. In addition, internationally there is a lack of registered factories capable of producing and delivering adequate equipment in a timely manner to protect HCWs on the frontlines.

#### Participating in meetings between various ETCs increased risk of infection

Many participants reported that communicating with other relevant organizations was important for international ETCs in Liberia, particularly for the new ETCs. Interviewees found that joining in-person meetings held by NECC was the only approach to communicate between ETCs from different countries. However this practice was not appropriate as gathering staff from multiple ETCs in one location could easily have resulted in infection. Using web based platforms for HCWs in ETCs to communicate with each other would be a safer option.

#### The CMMT lacked a member proficient in health diplomacy and foreign affairs

Interview participants thought their lack diplomatic skills partly contributed to the delayed delivery of supplies from China, and their inability to efficiently apply for supplies from other organizations or local governments.

## Discussion

HCWs working in West African ETCs had high rates of EVD infection. Since the start of the Ebola outbreak, a total of 881 confirmed HCW infections were reported in Guinea, Liberia, and Sierra Leone, and there have been 513 reported deaths [33]. Yet despite these high rates of infection, the CMMT members working in the Chinese ETCs had a zero infection rate. Since this study did not use case control, it is impossible to completely rule out the possible role of outside mitigating factors. However, the CMMT deployment started in September, during the peak of the pandemic when there were approximately 120 confirmed cases among HCWs [34]. During the same period of time that the CMMT was in Liberia, rates of EVD infection among HCWs continued to occur at high levels (comprising 3% of the overall cases), strongly supporting our claim that the particular procedures undertaken by the CMMT protected the HCWs [34]. Furthermore, HCWs in West African ETCs had high rates of mental distress during the outbreak [35]. In contrast, a study on mental distress among Liberian medical staff working at the Chinese ETC found that mental distress among study participants was not as serious [22].

This study found two distinctive health protection strategies were employed to ensure the health of the CMMT. Firstly, the strategy for health protection was comprehensive and multidisciplinary. Protecting staff from Ebola infection was the key issue, but the CMMT also received mental health care, as strong mental health was considered important to strengthening resistance to infection. Measures taken to promote mental health, and provide comfortable living and working environments were considered an important part of the health protection policies and procedures. Health protection was included in all phases of the mission (before-, during- and post-deployment), rather than just during the deployment phase when CMMT members were under direct threat of EVD infection. The risk factors varied during the different phases, and protection measures were adjusted accordingly. Measures before the mission were necessary to develop and strengthen the capability of the team to undertake the mission, both physically and psychologically.

Secondly, the CMMT undertook unique measures to minimize the HCWs exposure to Ebola. HCWs are mainly infected with Ebola through nosocomial transmission [36]. One of the causes of this nosocomial transmission is the difficulty in the clinical recognition of Ebola due to its nonspecific and nonpathognomonic presenting symptoms. The lack of diagnostic capabilities complicates prevention efforts at the beginning of an outbreak [37]. Therefore, provision high quality PPE is critical to prevent infection while providing care to patients [37]. However, once Ebola was diagnosed, an insufficient supply of PPE, and inadequate training on PPE use increased EVD infection among HCWs [37]. The modified PPE used by the CMMT was found to be much more effective and comfortable by both Chinese and Liberian staff [38]. In order to prevent future problems with PPE, there needs to be a certain number of registered factories capable of producing adequate equipment. Correct preparation of international medical teams was another key factor in the successful Ebola response. Training medical teams on the proper use of the equipment is essential. Training camps should be equipped to accurately simulate the conditions in the emergency location to properly prepare teams for their work in the field.

The physical design of the ETC was an important aspect of the CMMT’s protection. Most of the newly established or provisionally transformed ETCs comprise a contaminated “red” zone and a non-contaminated “green” zone to meet the standard WHO recommendation [39]. However, the Chinese ETC developed the aforementioned system which included “Three Zones and Two Routes”, this system helped to prevent disease transmission within the ETC. Physical and emotional exhaustion often causes HCWs to make mistakes when removing their PPE, such as following the impulse to wipe away sweat, which may increase the chance of inadvertent exposure to bodily fluids on the outside of the PPE [37]. Although, the Interim Guidance released in September 2014 by WHO required HCWs to be observed while placing or removing PPE to prevent mistakes [39], the three-level safety supervision program, and the video monitoring system used in the Chinese ETC provided better supervision and more timely guidance when mistakes were made [23]. This stricter protective system had a significant impact on infection prevention, and was recommended for use in future emergent infectious disease control.

International standard protocols are necessary to improve the level of global health responses. Although considerable progress has been made in the establishment of common standards and protocols for use in responding to disasters, [40, 41] and essential public health principles for management of all infectious diseases were proposed during the Ebola outbreak [42], there is still a lack of comprehensive international emergency response standards and protocols on infectious disease outbreaks. Comprehensive guidelines to help international organizations understand the diseases, and geographical and social conditions in their countries of deployment are essential. Furthermore, these documents should be easily accessible, for example a special section on epidemics on the WHO website could be established to publish and update the material.

It is essential for systematic risk assessments to be carried out by global health professionals when medical teams respond to international health emergencies. The CMMT drew on their previous experience from SARS in China to anticipate risks they might encounter, however this was not entirely adequate as West Africa is vastly different from China. In order to play a significant role in responding to global health emergencies, China needs to strengthen its knowledge and experience in global health. The Chinese government needs to pay greater attention global health research, and cultivate global health leaders.

This study had the following limitations. Firstly, this study only utilized in-depth interviews to collect data. Participant responses can be influenced by the interviewer, for example interviewers with poor interview skills may ask leading questions, or influence interviewee’s responses in other ways. However, the researchers conducting the interviews received training on proper interview technique, and we used semi-structured open-ended questions to mitigate this limitation. Secondly, this study collected data 5 months after the CMMT had returned to China. This time delay may have resulted in some recall bias on behalf of the participants. We tried to recruit at least two team members with similar roles and responsibilities in the CMMT to increase the validity of data.

## Conclusions

This study is the first of its kind to analyse the multiple risk factors HCWs were exposed to throughout their mission in Liberia using the bio-psycho-social model, as well as to comprehensively examine the policies and procedures used to protect the overall health of HCWs. The experiences of the CMMT compared to other HCWs would strongly suggest that the comprehensive and multidisciplinary health protection strategies provided substantial protection against Ebola, other infectious diseases, and psychological stressors. However, this study also identified a number of challenges. With an increasing number of emerging and re-emerging diseases, international medical teams will often be deployed to respond to global health emergencies around the world. In order to better protect the health of these medical teams, the development of protective health measures needs to be prioritized. These measures should include: capable command systems; effective coordination mechanisms; adequate equipment; comprehensive training for medical teams; investment in developing global health professionals; and placing an emphasis on research related to health protection for medical teams. The successes and challenges experienced by the medical team in the Chinese ETC have implications for future practice and research. The experiences summarized in this study are derived from a medical team deployed in a resource-limited environment, and might have significant implications for future global health emergencies in similar settings.

## Additional file

Additional file 1: Multilingual abstracts in the five official working languages of the United Nations. (PDF 288 kb)

## Abbreviations

CCHF: Crimeane Congo hemorrhagic fever
CDC: Center of Disease Control and Prevention
CMMTs: Chinese military medical teams
ETCs: Ebola treatment centers
EVD: Ebola virus disease
HCWs: Health care workers
HCWs: Healthcare workers
NECC: National Ebola Command Center
PLA: People’s Liberation Army
PPE: Personal protective equipment
SARS: Severe acute respiratory syndrome
VHFs: Viral hemorrhagic fevers
WHO: World Health Organization
