# Supplementary material for: Experiences and challenges in the health protection of medical teams in the Chinese Ebola treatment center, Liberia: a qualitative study
Source: Infect Dis Poverty. 2018 Aug 16;7:92. doi: 10.1186/s40249-018-0468-6 (PMC6103862; doi:10.1186/s40249-018-0468-6)
Supplement: Supplementary file 1 — Multilingual abstracts in the five official working languages of the United Nations. (PDF 288 kb) [file 40249_2018_468_MOESM1_ESM.pdf]

Translation of the abstract into the five official working languages of the United Nations

الخبرات والتحديات في مجال الحماية الصحية للفرق الطبية في مركز علاج الإيبولا الصيني في ليبيريا: دراسة نوعية

يينغ لي، هوان وانج، شو روي جين، شيانغ لي، ميشيل بيندر، كاي بينغ سونغ، شينغ لان تانغ، شينغ لن تانغ، جيا تساو، هاو وو
ووانغ-جوي وانغ

نبذة مختصرة
الإطار العام: يعمل مقدمو الرعاية الصحية على خط المواجهة في مكافحة الأمراض المعدية، ونتيجة لذلك يكونون عرضة لخطر العدوى. خلال تفشي الإيبولا في غرب أفريقيا 2014-2015 ، أصيب العديد من العاملين في مجال الرعاية الصحية بالإيبولا، وكانت إصابات بعضهم قاتلة. ومع ذلك، لم يصب أي من أعضاء الفريق الطبي الصيني لمكافحة الإيبولا، الذي شارك في تقديم الرعاية الطبية الحيوية في ليبريا. تهدف هذه الدراسة إلى معرفة كيف تم تفادي الإصابة بعدوى الإيبولا.
طرق العمل: تم جمع البيانات من خلال 15 مقابلة معمقة مع عناصر من جيش التحرير الشعبي في الصين الذي كونوا الفريق الطبي المشرف على مركز علاج الإيبولا في الفترة الممتدة من أكتوبر 2014 إلى يناير 2015 في ليبيريا. تمت معالجة البيانات باستخدام إطار منهجي للتحليل.
النتائج: اكتشفت هذه الدراسة العديد من المخاطر الحيوية و النفسية والاجتماعية والسلوكية التي تهدد بشكل مباشر أو غير مباشر صحة الفريق الطبي العامل في مركز علاج الإيبولا الصيني. وشملت هذه المخاطر الإجهاد الاجتماعي والعاطفي الناجم عن: (1) الابتعاد عن دائرة العلاقات الأسرية والاجتماعية المألوفة؛ (2) صعوبة التكيّف مع ثقافة مختلفة؛ (3) والشعور بالقلق بسبب الاضطرابات الاجتماعية والسياسية في ليبيريا. كان تعرض المرضى والعمال المحليين لخطر فيروس إيبولا، ناهيك عن سوء استخدام وسائل الحماية الشخصية بسبب التعب، أحد المخاطر الكبيرة الأخرى. من بين المخاطر الأخرى التي تم تحديدها: (1) نقص الإمدادات؛ (2) نقص العاملين الصحيين المدربين؛ (3) استهلاك الأغذية والمياه الملوثة؛ (4) وساعات العمل الطويلة. تم بذل جهود شاملة طيلة المدة التي استغرقتها المهمة، للتعامل مع هذه المخاطر. تم اتخاذ كل التدابير لمنع تعرض الفريق الطبي لفيروس الإيبولا، ولتهيئة بيئة عمل آمنة ومريحة للفريق الطبي. كان هناك العديد من التحديات في سبيل الحفاظ على السلامة الصحية للفريق، مثل القدرة المحدودة لنظام معالجة الطوارئ (طريقة موحدة لمواجهة حالات الطوارئ ومراقبتها وتنسيق الجهود المبذولة في ذلك الإطار)، وعدم وجود بروتوكولات دولية شاملة تشرح كيفية التعامل مع حالات الطوارئ المتعلقة بالأوبئة المعدية.
الخلاصة: أثبتت التدابير الشاملة والمتعددة الاختصاصات المستخدمة لحماية صحة الفريق الطبي نجاحها حتى في بيئة ليبيريا المحدودة الموارد. يمكن للمنظمات العالمية العاملة في المجال الصحي أن تتعلم دروساً قيمة من هذه التجربة التي يمكنها أن ترفع من مستوى سلامة العاملين في مجال الرعاية الصحية في حالات الطوارئ في المستقبل. من ضمن هذه الدروس المستفادة نذكر: ضرورة إنشاء أنظمة قيادة فاعلة؛ ضرورة بعث آليات تنسيق فعالة؛ ضرورة توفير المعدات المناسبة؛ ضرورة توفير التدريب المناسب للفرق الطبية؛ ضرورة الاستثمار في رفع كفاءة العاملين بالمجال الصحي في المنظمات العالمية؛ وضرورة تحسين مستوى البحوث حول طرق حماية العاملين في مجال الرعاية الصحية.

Translated from English version into Arabic by Maher Abassi and Sophie Chammas, through

TRANSLATORS
WITHOUT BORDERS

中国在利比里亚抗击埃博拉医疗队健康维护的经验和挑战:定性研究

李颖，王欢，晋旭锐，李翔，Michelle Pender，宋彩萍，汤胜蓝，曹佳，吴昊，王云贵

摘要

引言：医务人员作为传染病控制一线人员，面临更容易被感染的危险。在 2014-2015 年西非埃博拉疫情中，很多医务人员感染并死于埃博拉。然而，在利比里亚中国埃博拉防治中心实现了医务人员零感染的目标。该研究的目的是分析该医疗对健康维护的经验，总结存在的挑战。
方法：通过对 15 位在利比里亚中国埃博拉防治中心的一线医务人员进行个人深入访谈收集资料，采用国际上通用的框架分析法对资料进行分析。
结果：该研究发现在在利比里亚中国埃博拉防治中心的一线医务人员面临着复杂的社会-心理-生物危险因素。医疗队采取多种措施维护大家的健康，包括预防感染埃博拉病毒的医疗卫生措施，通过社会学和卫生外交措施为医疗队提供健康安全的工作和生活环境。但是，医疗队也遇到很多挑战，如应急指挥体系的不力，国际上缺乏应对传染病引起的公共卫生问题的指南等。
结论：在利比里亚中国埃博拉防治中心的医疗队面临很多健康危险因素，面临着感染埃博拉病毒和各种心理健康问题的威胁。但是第三军医大学采取的多学科综合的健康维护策略对医疗队的健康维护起到很好的保护作用，这些措施在资源比较贫乏的条件下也具有可行性。相关经验教训可为以后类似的全球性突发公共卫生事件处置过程中医务人员健康维护提供重要借鉴。

Translated from English version into Chinese by Ying Li

Expérience et difficultés de la protection de la santé des équipes médicales au Centre chinois de traitement de la fièvre d'Ébola au Liberia : étude qualitative

Ying Li, Huan Wang, Xu-Rui Jin, Xiang Li, Michelle Pender, Cai-Ping Song, Sheng-Lan Tang, Sheng-Lan Tang, Jia Cao, Hao Wu et Yun-Gui Wang

Résumé

Contexte : En première ligne dans la lutte contre les maladies infectieuses, les professionnels de la santé se trouvent exposés, de ce fait, à un risque infectieux important. Ils ont été nombreux à contracter la fièvre d'Ébola au cours de l'épidémie de 2014-2015 en Afrique de l'Ouest, et certains y ont succombé. Pourtant, aucun membre de l'équipe médicale anti-Ébola chinoise déployée au Liberia n'a été infecté. La présente étude a pour but de comprendre comment ce taux zéro d'infection a pu être obtenu.
Méthodes : Au moyen de 15 entretiens approfondis, nous avons recueilli des informations auprès des membres de l'équipe médicale de l'Armée populaire de libération chinoise détachés dans le Centre chinois de traitement de la fièvre d'Ébola au Liberia entre octobre 2014 et janvier 2015 Ces données ont été analysées au moyen d'une analyse du cadre logique.
Résultats : L'étude a identifié de nombreux facteurs de risque biologiques, psychologiques, sociaux et comportementaux qui menaçaient, directement ou indirectement, la santé de l'équipe médicale du Centre chinois de traitement de la fièvre d'Ébola. Ces facteurs incluaient le stress social et émotionnel causé par (1) la distension des liens familiaux et sociaux, (2) l'adaptation à une culture différente et (3) l'inquiétude suscitée par l'agitation sociale et politique au Liberia. Le risque de contamination par les patients et les collègues libériens et les erreurs d'utilisation des équipements de protection individuelle dues à la fatigue étaient également des facteurs de risque majeurs. S'y

ajoutaient aussi(1) le manque de fournitures, (2) le manque de personnel médical qualifié, (3) l'exposition à des aliments et de l'eau contaminés et (4) les longues journées de travail. Tout au long de la mission, des mesures ont été prises sur tous ces axes pour atténuer les risques. Tout a été fait pour empêcher l'exposition de l'équipe au virus Ébola et créer un environnement de travail et de vie sûr et confortable. Le maintien de la sécurité sanitaire de l'équipe a rencontré de nombreuses difficultés, comme la capacité limitée du système de commandement d'urgence (approche standardisée du commandement, du contrôle et de la coordination d'une réponse d'urgence) et l'absence de protocoles internationaux complets pour faire face aux pandémies émergentes.

Conclusions : Les mesures complètes et multidisciplinaires déployées pour protéger la santé de l'équipe médicale se sont avérées efficaces même dans le contexte de ressources limitées du Liberia. La communauté médicale mondiale pourrait tirer de cette expérience de précieux enseignements pour améliorer la sécurité des professionnels de la santé lors des crises futures : création de systèmes de commandement fonctionnels, mise en place de mécanismes de coordination efficaces, fourniture d'équipements adéquats, formation des équipes médicales, investissements dans la formation de professionnels de la santé globale et amélioration de la recherche sur les moyens de protéger les professionnels de la santé.

Translated from English version into French by Suzanne Assenat and Charlote Giovan, through

TRANSLATORS
WITHOUT BORDERS

Опыт и проблемы охраны здоровья медицинского персонала Китайского центра по лечению Эболы в Либерии: качественное исследование

Ин Ли, Хуань Ван, Сюй-Жуй Цзинь, Сян Ли, Мишель Пендер, Цай-Пин Сун, Шэн-Лань Тан, Цзя Цао, Хао У и Юнь-Гуй Ван

Аннотация

Общие сведения: Медицинские работники находятся в авангарде борьбы с инфекционными заболеваниями и потому подвержены высокому риску заражения. Во время вспышки Эболы в Западной Африке в 2014-2015 многие медицинские работники заразились, некоторые умерли. При этом никто из медицинского персонала Китайского центра по лечению Эболы, направленного в Либерию для оказания необходимой медицинской помощи, заражен не был. Цель настоящего исследования – выяснить, как им удалось добиться нулевого показателя инфицирования.

Методы: Данные были собраны в ходе 15 подробных опросов медицинского персонала Народно-освободительной армии Китая, работавшего в Китайском центре по лечению Эболы в Либерии с октября 2014 по январь 2015. Данные были подвергнуты системному рамочному анализу.

Результаты: Настоящее исследование выявило многочисленные биопсихосоциоповеденческие факторы риска, которые прямо или косвенно угрожали

здоровью медицинского персонала, работавшего в Китайском центре по лечению Эболы. В число этих факторов входил социальный и эмоциональный стресс, вызванный: (1) нарушением семейных и социальных связей; (2) адаптацией к чужой культуре и (3) беспокойством по поводу социальных и политических волнений в Либерии. Кроме того, существенными факторами риска являлись контакты с носителями вируса из числа пациентов и местных коллег, а также неправильное использование средств индивидуальной защиты из-за переутомления. Также были выделены следующие факторы риска: (1) нехватка медикаментов; (2) отсутствие квалифицированного медицинского персонала; (3) употребление зараженной пищи и воды и (4) большая продолжительность рабочего дня. Для минимизации воздействия этих факторов на протяжении всей миссии прилагались значительные усилия. Были предприняты всевозможные меры для предотвращения заражения медицинских работников вирусом Эбола и создания им безопасных и комфортных условий работы и жизни. Охрану здоровья персонала осложняло множество проблем, например, ограниченность возможностей системы экстренного реагирования (стандартизированный подход к управлению, контролю и координации реагирования на чрезвычайные ситуации) и отсутствие подробных международных протоколов борьбы с пандемиями новых инфекционных заболеваний.
Выводы: Масштабные и разноплановые меры по охране здоровья медицинского персонала в Либерии дали положительные результаты даже в условиях ограниченности ресурсов. Мировое медицинское сообщество может извлечь из этого опыта ценные уроки, которые помогут сделать условия работы медицинского персонала в ходе будущих чрезвычайных ситуаций более безопасными. К таким мерам относятся: создание действенных систем реагирования; внедрение эффективных механизмов координации; предоставление необходимого оборудования; обучение медицинского персонала; инвестирование в повышение профессионального уровня работников систем здравоохранения во всем мире и улучшение качества исследований в области охраны здоровья медицинского персонала.

Translated from English version into Russian by Liudmila Tomanek and Anna Romanenko, through

TRANSLATORS
WITHOUT BORDERS

Experiencias y desafíos en la protección de la salud de los equipos médicos en el Centro Chino de Tratamiento del Ébola, Liberia: un estudio cualitativo

Ying Li, Huan Wang, Xu-Rui Jin, Xiang Li, Michelle Pender, Cai-Ping Song, Sheng-Lan Tang, Sheng-Lan Tang, Jia Cao, Hao Wu y Yun-Gui Wang

Resumen

Antecedentes: Los trabajadores de la salud se encuentran a la vanguardia en la lucha contra las enfermedades infecciosas y, como resultado, corren un alto riesgo de infección. Durante el brote de Ébola 2014-2015 en África occidental, muchos trabajadores de la salud contrajeron el Ébola,

algunos de ellos con consecuencias fatales. Sin embargo, ningún miembro del equipo médico chino Anti-Ébola, desplegado para proporcionar atención médica vital en Liberia resultó infectado. Este estudio pretende comprender cómo se logró esta tasa de cero infección.

Métodos: Los datos fueron recopilados a través de 15 entrevistas exhaustivas con participantes del equipo médico del Ejército Popular de Liberación de China que operó el Centro Chino de Tratamiento del Ébola desde octubre de 2014 hasta enero de 2015 en Liberia. Los datos fueron analizados utilizando un análisis de marco sistemático.

Resultados: Este estudio encontró numerosos factores de riesgo biopsico-socio-conductuales que directa o indirectamente amenazaron la salud del equipo médico que trabajó en el Centro Chino de Tratamiento del Ébola. Estos factores incluyen el estrés social y emocional causado por: (1) la interrupción de las redes familiares y sociales; (2) la adaptación a una cultura diferente; (3) y la ansiedad por los disturbios sociales y políticos en Liberia. La exposición al Ébola por parte de pacientes y compañeros de trabajo locales, y el uso incorrecto del equipo de protección personal debido a la fatiga fue otro factor de riesgo importante. Otros factores de riesgo identificados fueron: (1) escasez de suministros; (2) falta de personal de salud capacitado; (3) exposición a alimentos y agua contaminados; (4) y largas horas de trabajo. Se realizaron esfuerzos integrales durante toda la misión para mitigar estos factores. Se tomaron todas las medidas para evitar la exposición del equipo médico al virus del Ébola y para proporcionar al equipo médico ambientes de trabajo y de vida seguros y cómodos. Hubo muchos desafíos para mantener la seguridad sanitaria del equipo, como la capacidad limitada del sistema de comando de emergencia (el enfoque estandarizado del comando, control y coordinación de una respuesta de emergencia), y la falta de protocolos internacionales integrales para tratar las pandemias emergentes de enfermedades infecciosas.

Conclusiones: Las medidas integrales y multidisciplinarias empleadas para proteger la salud del equipo médico demostraron ser exitosas incluso en el contexto de los recursos limitados de Liberia. La comunidad sanitaria global puede aprender valiosas lecciones de esta experiencia que podrían mejorar la seguridad de los trabajadores de la salud en futuras emergencias. Estas lecciones incluyen: establecer sistemas de comando capaces; implementando mecanismos efectivos de coordinación; proporcionar el equipo adecuado; proporcionar entrenamiento para equipos médicos; invertir en el desarrollo de profesionales de la salud global; y mejorar la investigación sobre formas de proteger a los trabajadores de la salud.

Translated from English version into Spanish by Guadalupe Barua and Maria Paula Gorgone, through

TRANSLATORS
WITHOUT BORDERS
